# Retraction: Secreted Frizzled-Related Protein 4 Inhibits Glioma Stem-Like Cells by Reversing Epithelial to Mesenchymal Transition, Inducing Apoptosis and Decreasing Cancer Stem Cell Properties

**DOI:** 10.1371/journal.pone.0268372

**Published:** 2022-05-05

**Authors:** The PLOS ONE Editors

After publication of this article [1], concerns were raised about data reported in Figs 1c, 3, 4, and 5.

An Academic Editor advised that the flow cytometry experiments reported in Figs 1c, 3 and 5 were not conducted or analysed correctly and did not include key controls. In light of these issues, statements claiming an enrichment of cancer stem cells based on FACS analysis (CD133+ cells; Fig 1C) and statements claiming impacts of drug treatments on CD133+ populations (Fig 5) are not supported.

The Academic Editor further advised that the plots shown in Fig 4 do not report MitoProbe apoptosis assay results as per community standards, it is unclear what the plots represent, and they do not support the claims made in the article about apoptosis levels in treated versus untreated GSCs. It was further raised in post-publication discussions that this fig does not report a statistical analysis of the results.

The corresponding author noted that the original data underlying Figs 1c, 4, and 5 are not available.

In reviewing this matter, it came to light that the cell lines used in this study may be misidentified, according to information available on the Expasy database, [2–3]. Hence the relevance of the results for gliomas and glioma stem cells is in question.

Note, the primary data are not provided with the article, though the Data Availability Statement says, “All relevant data are within the paper and its Supporting Information files.”. The corresponding author noted that underlying data are available for Figs 1a, 2c, 7a and 9a-c, but are not available for Figs 1b and c, 2a and b, 3, 4, 5, 6, 7b, 8, 10 and S1-3.

The above concerns have significant implications for the reliability of the article’s main results and conclusions, and indicate that the article does not meet *PLOS ONE*’s third publication criterion or comply with the PLOS Data Availability policy. Therefore, the *PLOS ONE* Editors retract this article. The *PLOS ONE* Editors apologise that these concerns were not addressed prior to publication of this article.

SW did not agree with retraction. FA did not directly comment on their position on the retraction decision. BG, AD, and MM could not be reached.
